# Mobile Apps for Eye Care in Canada: An Analysis of the iTunes Store

**DOI:** 10.2196/mhealth.7055

**Published:** 2017-06-14

**Authors:** Alexander Rodin, Aviv Shachak, Aaron Miller, Vladimir Akopyan, Nataliya Semenova

**Affiliations:** ^1^ TorontoEyeExam.com Oakville, ON Canada; ^2^ Department of Ophthalmology Faculty of Fundamental Medicine Lomonosov Moscow State University Moscow Russian Federation; ^3^ CanMedApps Inc. Thornhill, ON Canada; ^4^ Institute of Health Policy, Management & Evaluation and The Faculty of Information University of Toronto Toronto, ON Canada; ^5^ ELLICSR Health, Wellness & Cancer Survivorship Centre Toronto General Hospital University Health Network Toronto, ON Canada

**Keywords:** mobile applications, mobile phone, ophthalmology, optometry

## Abstract

**Background:**

Mobile phone screens can facilitate stimulation to various components of the visual system and many mobile apps are accepted as a means of providing clinical assessments for the oculo-visual system. Although many of these apps are intended for use in clinical settings, there is a growing number of apps in eye care developed for self-tests and eye exercises for lay people. These and other features, however, have not yet been well described.

**Objective:**

Our objective was to identify, describe, and categorize mobile apps related to eye care that are available to users in the Canadian iTunes market.

**Methods:**

We conducted an extensive search of the Apple iTunes Store for apps related to eye care. We used the terms “eye,” “eye care,” “vision,” and “eye test” and included apps that are targeted at both lay people and medical professionals. We excluded apps whose primary function is not related to eye care. Eligible apps were categorized by primary purpose, based on how they were described by their developers in the iTunes Store.

**Results:**

Our search yielded 10,657 apps, of which 427 met our inclusion criteria. After removing duplicates, 355 unique apps were subject to further review. We assigned the eligible apps to three distinct categories: 39/355 apps (11.0%) were intended for use by medical professionals, 236 apps (66.5%, 236/355) were intended for use by lay people, and 80 apps (22.5%, 80/355) were intended for marketing eye care and eye-care products. We identified 9 subcategories of apps based on the descriptions of their primary functions. Apps for medical professionals fell into three subcategories: clinical calculators (n=6), clinical diagnostic tools (n=18), and education and networking apps for professionals (n=15). Apps for lay people fell into four subcategories: self-testing (n=153), eye exercises (n=30), patient tools and low vision aids (n=35), and apps for patient education (n=18). Mixed-use apps (n=80) were placed into two subcategories: marketing of individual practitioners or eye-care products (n=72) and marketing of multiple eye-care products or professional services.

**Conclusions:**

The most extensive subcategory pertaining to eye care consisted of apps for use by lay people, especially for conducting self-tests (n=236). This study revealed a previously uncharacterized category of apps intended for use by doctors and patients, of which the primary goal is marketing of eye-care services and products (n=80).

## Introduction

In recent years, there has been growth in apps for both lay people and clinicians [1]. It is estimated that by the year 2018, half of all mobile phone users will download at least one health-related app [2]. Emerging mobile-based technologies can affect the eye-care market substantially. From the perspective of a lay person, this facilitates innovative communication channels with clinicians based on questions and images, while also empowering lay people with self-testing methods in the palms of their hands. Self-testing apps may be particularly useful for patients living in remote and low-resource areas [3]. Mobile technologies also provide a new framework for the digital connectivity of ophthalmic diagnostic devices for eye-care professionals, supporting real time decision-making, streamlining diagnostic processes, and opening new modalities for business practices and enterprise promotion.

Extensive research has evaluated mobile technologies and their readiness for clinical practice, including the evaluation of mobile color vision tests [4], visual fields tests (Amsler grid) [5], and mobile phone add-ons that convert the camera of the phone into a miniature anterior segment and retinal camera [6]. Apps for home monitoring and self-testing, including the myVision Track app, were cleared by the FDA [7].

Recent reviews on ophthalmologic apps found that they were largely clinician-oriented. It was suggested by Chhablani et al [2] that mobile apps for eye care be divided into five main categories: patient education, patient self-testing, patient visual aids, patient records and administrative tools, and programs supporting emerging hardware tools. Other studies [8] suggest that these apps be placed within five distinct categories, including patient assessment tools, patient education tools and visual aids, patient records, health care profession education, and reference. This study also suggests the addition of a broad category of “multiple function” apps. It is important to consider that in such a dynamic and volatile marketplace as that of mobile apps, technologies can change rapidly, thereby affecting how popular they may be and what their patterns of use may include.

The purpose of the study was to identify, describe, and categorize eye-care apps available to users of the Canadian Apple iTunes Store.

## Methods

An extensive search of the Apple iTunes Store was performed for apps that related to eye care, following the Preferred Reporting Items for Systematic Reviews and Meta-Analyses (PRISMA) guidelines [9]. A recent study on the evaluation of app quality [10] revealed that iOS does not traditionally support older apps once a newer version of the operating system becomes available. This contrasts with Google Android, where older apps may remain available to users in the marketplace unless they are manually removed by the developer. To avoid the number of outdated apps that were no longer supported by the developer as well to reduce biases resulting from different app ranking algorithms, only the iOS market sample was included in the study.

The search hedge for this study was performed between January and February 2016 and included the terms “eye,” “vision,” “eye test,” and “eye care.”

Conventionally, eye care has been defined as the prevention or minimization of threats to the eye or to visual integrity [11]. Per the World Health Organization (WHO) [12], a “health condition” is a complex interaction with contextual factors such as body structure, functions, participation in activities (including self-care), as well as environmental and personal factors. As eye health is a component of general health, we determined it necessary to include search terms pertaining to the eye-care domain. Our assumption was that the search terms “eye,” “vision,” “eye test,” and “eye care” are relevant and valid search terms for apps pertaining to eye care.

All apps that targeted medical professionals and patients were included in the study. We excluded apps that were not directly related to eye care, such as serious games or optical illusions. The results were screened and duplicates were removed. Eligible apps were coded based on the description provided by the developers in the iTunes Store and categorized by primary purpose based on their description. We did not apply a date range so as to include all apps that met the above described criteria.

## Results

**Figure 1:**
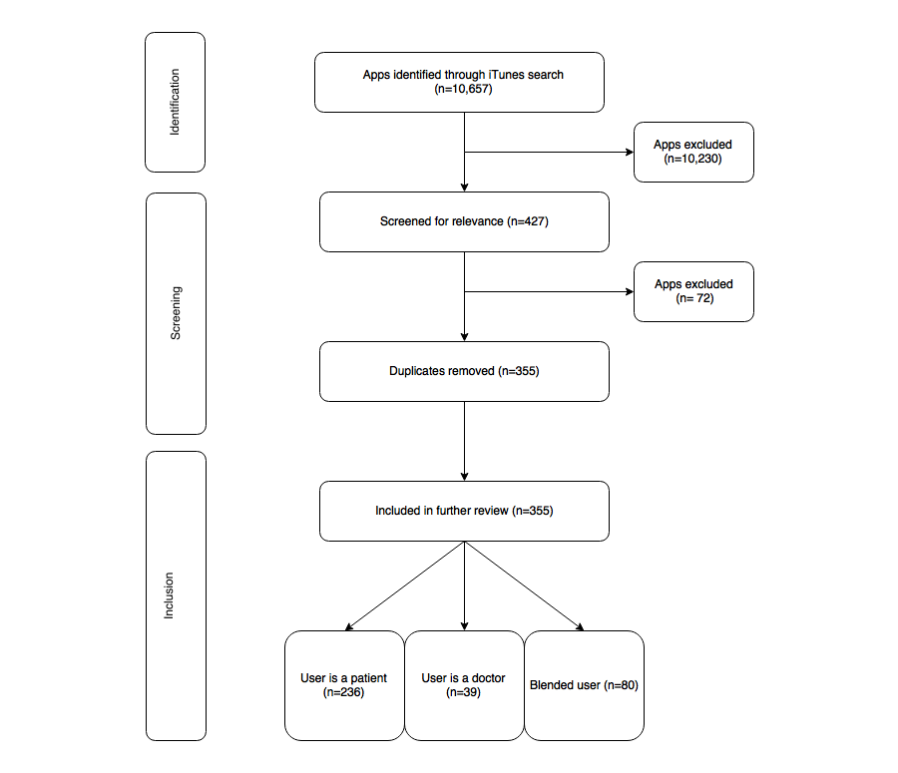
Systematic search for eye-care apps in the Canadian iTunes Store.

Our search identified 10,657 apps in total. Over 96.00% of the apps including those that were found using the search terms “eye,” “eye care,” “vision,” and “eye test” were excluded after the first round of screening as they were related to optical illusions, games, and utilities and did not meet our search criteria. Our inclusion identified 427 apps related to eye care in the iTunes Store. After removing duplicates, only 355 unique apps met our inclusion criteria and were therefore included in the review (Figure 1).

**Figure 2:**
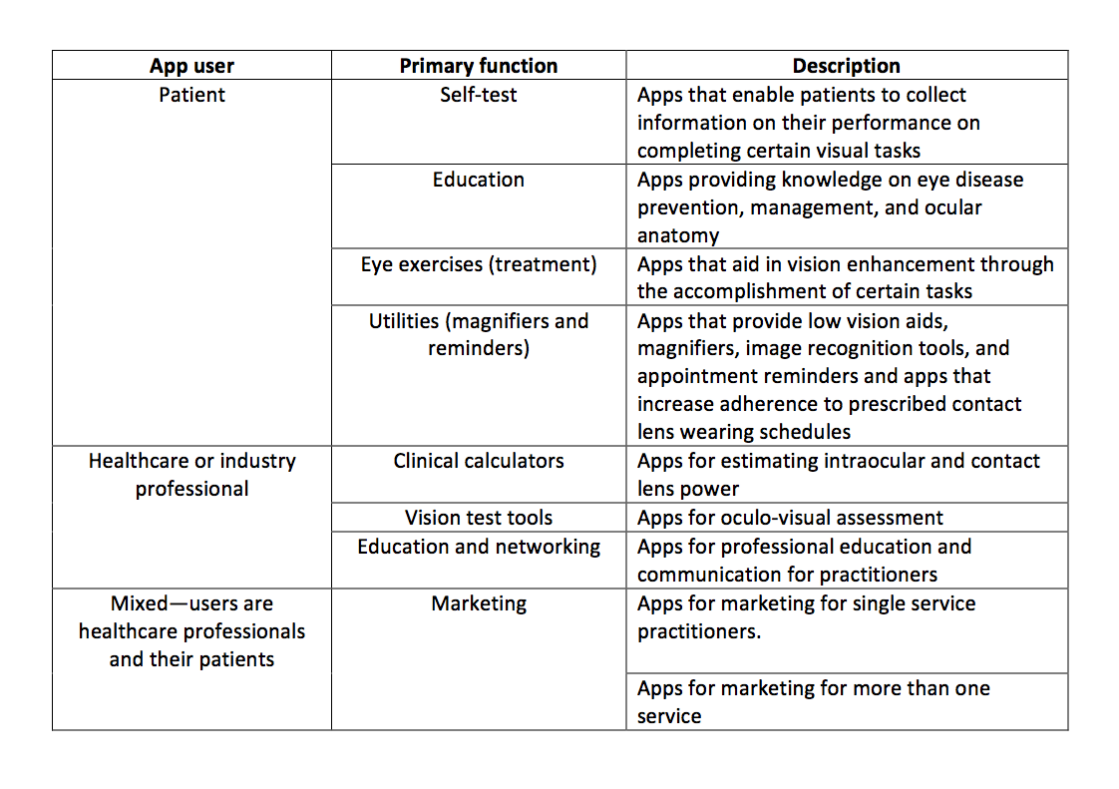
A classification of eye-care apps in the Canadian iTunes Store.

Based on the descriptions of the apps in the App Store, we assigned eligible apps to three primary categories: 236/355 apps (66.5%) intended for use by patients or lay people, 39/355 apps (11.0%) for use by medical professionals (n=39), and 80/355 apps (22.5%) with a blend of potential end-users. We conducted descriptive, qualitative analyses of these apps based on the descriptions provided by their developers to assist in developing these subcategories (Figure 2).

Four subcategories were described for patient-oriented apps, including self-tests, patient education tools, eye exercises or patient utilities, as well as low vision aids. The eye-care medical professional apps category is comprised of three subcategories, including clinical calculators, clinical diagnostic tools, and clinical education and networking apps. Finally, the mixed-use category consists of two subcategories, including apps for a single practitioner or product and those for multiple products or services.

The patient self-test subcategory consisted of apps that enabled patients to collect information pertaining to their performance after completing specific visual tasks. Apps for eye exercises included those that aimed to facilitate vision enhancement through the accomplishment of tasks. Patient education tools were those whose primary goal was providing information on eye disease prevention and maintenance as well as eye anatomy. A subcategory of patient utility tools was used for apps that provided low vision aids, magnifiers, image recognition tools, appointment reminders and apps that aimed to increase adherence to a prescribed contact lens-wearing schedule. The number of apps that fell into each of these subcategories was as follows: patient self-test (n=153), patient education apps (n=18), eye exercises (n=30), and patient utility, including low vision aids (n=35).

Apps for medical professionals (n=39) were divided into three subcategories. The first subcategory consisted of clinical calculators, including apps whose primary goal was assisting clinicians with quantitative analysis of data obtained from diagnostic instruments, such as intraocular lens calculation or vertex distance adjustment estimates used in contact lens fitting. The second subcategory consisted of clinical tools such as charts, figures, or instruments for oculo-visual assessment. The third category included medical professional education apps and apps intended to facilitate education, learning, communication, and collaboration for practitioners. Overall, there were 16 apps classified as clinical diagnostic tools for use by eye-care professionals.

Mixed use apps (n=80) were those that facilitated two-way communication between practitioners and their patients. The majority of these apps, however, were intended for the marketing of professional services or eye-care products, including appointment reminders or self-testing tools such as the Amsler grid. These apps were separated into two subcategories: 72 for marketing of single individual practitioner or eye-care products and 8 apps for lay people.

## Discussion

### Principal Findings

The largest group of apps in our study consisted of apps designed for patient self-testing (n=153). A literature review of studies related to the evaluation of mobile apps intended for exercises as a means of improving visual functions returned few studies. Recent studies relevant to apps available for eye care in the Apple Store [13] confirm that an estimated 37% of app developers included as a feature, consultation with a certified eye-care professional.

We conducted a supplementary PubMed search, but we were unable to identify relevant literature on the use of mobile apps for home-based vision therapy. We posit that one of the reasons for the paucity of studies in this domain is that from a Canadian medico-legal standpoint, eye exercises are considered to be vision therapy that according to the Optometry Act [14] must be administrated exclusively by health care professionals.

Patient education tools are presented in health apps for lay people as standalone applications (such as libraries, websites, and books) or as reference information materials provided to supplement other types of ocular health-related apps. We found 18 apps that were developed to be used by patients for expanding their knowledge and awareness about eye diseases, eye-health maintenance, and eye-disease prevention.

The patient utilities group consisted of 35 apps that were intended to help in the self-management of eye-care needs for visually impaired patients. In addition to magnifiers and apps that support object recognition, this category also includes apps that support adherence to a prescribed contacts lens wearing regimen. Another example includes a gesture recognition interface (currently in development), which has the potential to substantially increase engagement in users of apps who have low vision impairments [15].

A review by Meyer et al [16] posits the application of mobile technologies as visual aids; despite this, however, low vision aids remain underrepresented in studies. Authors on this topic describe the useful functionalities of mobile apps for patients with low vision, saying that they are capable of reading and communicating text fragments, recognizing products with barcodes, and enhancing spatial orientation for visually impaired patients using an integrated GPS. Overall, these functionalities of mobile phones will help visually impaired patients with spatial orientation, objects magnification, and reading a fine print [16].

Clinician-facing apps were described by Lord et al [17] and include clinical tests such as near vision cards, color vision plates, pupil gauges, pen and fluorescein lights, pediatric fixation targets, Amsler grids, Worth 4 Dot tests, accommodation targets, red desaturation tests, and an optokinetic nystagmus drum simulator. We classified 39 apps into this category in our study. Incidentally, many of these apps were assessed by eye-care professionals [16]. While one clinical study on Web-based applications for visual acuity and contrast sensitivity testing [18] included 104 participants, a strong limitation was that the application had been developed for desktop systems for telemedicine and not as a standalone mobile app.

In addition to these categories, we were able to identify categories of apps intended for use by eye-care professionals and develop a novel category—apps designed for marketing eye-care services and products. A category of mixed-use apps indicates that two-way communication between eye-care providers and their patients as end users is typical. In addition to traditional mobile telehealth apps for doctor-patient interactions previously reviewed by Nhavoto & Grönlund [19], it was found that the primary goal of these apps was the marketing of eye-care products and services. We expect this category of mixed-use apps to further grow and evolve as new offerings, such as the Apple HealthKit, that support communication between providers and patients as well as facilitate better integration of apps continue to develop.

In addition to marketing, we found that many of these apps are multifunctional and interactive. They include patient-centered tools such as doctor finder features, which help patients find eye-care clinics in their proximity, and product finders that promote online shopping for contact lenses and other eye-care products. This subcategory might also be classified as patient tools; however, their primary goals are consumer-oriented marketing and sales. We observe that this category has yet to be well described in the scientific literature. Previous studies might exclude this category from their search results to mitigate perceived commercial biases. However, in our view, these apps should not be excluded from consideration as they reflect a burgeoning market of eye-care apps in Canada.

It is evident that the number of eye care apps available for lay people is greater than those that are intended for medical professionals. We propose two factors that might influence this proposition. First, there is increasing demand for visual testing from lay people as the number of people afflicted by conditions of the eye in Canada is projected to increase by 4% by 2032 [20]. Second, new apps intended for clinical use require a designation as a medical device and must undergo a rigorous FDA or Health Canada certification process and this testing drives the costs for development and, therefore, for the end user. Though we did locate a few free clinically evaluated apps, such as SightBook [21], we found that those apps intended for clinical eye care were largely subject to the above described review process and could be priced in the Apple iTunes Store at well over CAD $100.

### Limitations

This study compiled data available in the Canadian iTunes Store in 2016 and represents only a snapshot of the very dynamic and vibrant environment of the mobile apps market. Our study design does not include apps from other app marketplaces for reasons explained in the methods section. Future research may elucidate this very important topic.

Although we attempted to categorize the apps based on their description in the App Store, the quality of the apps was not evaluated in a detailed fashion as suggested in the recent reviews on app quality assessment [22]. A longitudinal study that observes the growth and proliferation of optometric apps over time may also be similarly beneficial.

### Conclusions

While mobile apps for eye conditions, monitoring, visual aids, and use by providers are growing substantially, our search for apps related to the eye and eye care in the Apple iTunes Store found that only 4.00% of the apps are, in fact, intended for use in eye care. Among these apps, self-testing represents the largest category (66.5%), yet few are properly evaluated. The wide proportion of mixed-use apps (22.5%) focused on the marketing of eye-care products and services may support the argument that the continued development of health-related apps is compelled by sustained growth in this industry.
